# Reports on Hospitals of the United Kingdom

**Published:** 1914-05-30

**Authors:** Henry Burdett


REPORTS ON

### Hospitals of the United Kingdom.

By SIR HENRY BURDETT, K.C.B., K.C.V.O.

SERIES III.

### NORTH EVINGTON POOR-LAW INFIRMARY, LEICESTER.

This infirmary was established in 1905, and contains 500 beds, of which, on an average, 471 are occupied. The number of in-patients varies, being about 1,800 in the year on the average, which shows that a number of beds are occupied by chronic patients or by indigent cases which have overflowed from the workhouse, situate in the town. Of the 400 beds available for acute cases, we understand that upwards of 135 are occupied by mental cases and epileptics, so that the actual number of beds available for sick and injured patients is about 250 at present.

We visited this infirmary on March 9 and were received by the Chairman of the Board, the Chairman of the House Committee, and the Matron, Miss Masters. We made a thorough inspection of the hospital portion of the infirmary, under the conduct of one of the assistant medical officers, Dr. Fergus, the senior medical officer, being away.

### The Buildings and Equipment.

This infirmary has many attractive features, most of which are excellent. It cannot, however, be regarded as a hospital or Poor-Law infirmary within the proper and technical meaning of either term, because it is not at present so organised, staffed, or equipped, nor does it contain an operation theatre. The day-room, which has been temporarily denominated the theatre, is not sufficiently well lighted, nor is it heated so as to enable important operations to be performed there. The wards are well proportioned, and the sanitary and bathing arrangements and the equipment throughout are remarkably good; some of the furniture, notably the ward tables and dressers, has been constructed from an excellent model which, we understand, was designed by the late Matron, Miss Clark. We have often been asked to recommend a table of this description which would be suitable for one of the smaller hospitals, where the necessary stores, dressings, poisons, lotions, and other articles used in the ordinary course could be placed, upon a classified plan to facilitate the working of the ward and the orderly arrangement of these most necessary articles. Part of the bedding was excellent, and although the blankets were poor in quality we were assured that extra blankets, as well as hot bottles, were available for the use of patients who desired to have them. Whoever was responsible for the design and plan of this Poor-Law hospital deserves high credit, and if and when the existing buildings, excellent as they are, and excellently maintained by the intelligent action of the Board of Guardians, are made efficient and capable of providing modern, up-to-date hospital treatment and nursing for the poorer inhabitants of Leicester, the North Evington Poor-Law Infirmary should, under the direction of an experienced medical superintendent—with an adequate salary commencing at not less than £500 a year in addition to house allowance and service, etc.—speedily become one of the most attractive and useful Poor-Law hospitals in the country.

We understand there is a section of the ratepayers who have maintained throughout that the money expended on the present buildings has been excessive and that this expenditure ought never to have been incurred. As a matter of fact, this criticism is partly justified by the circumstance that up to the present time North Evington has never been an infirmary, with all the equipment, staff, and efficiency of a modern, up-to-date hospital such as the money expended demands. On the other hand, the criticism cannot be upheld if the best interests of the poorer citizens of Leicester are to be conserved as they ought to be conserved, having regard to the high standard and excellent principles which have made all other medical institutions and the principal kindred buildings in this town examples, in most respects, of what these institutions ought to be. For Leicester is a city where the inhabitants wisely act in cordial co-operation, with administrative results that make for efficiency and may be taken as a good type for the imitation of kindred communities up and down the country. How it has happened that so excellent a plan and so large an expenditure should have found acceptance with those responsible for the administration, upkeep, and efficiency of a great Poor-Law hospital such as North Evington ought to be to-day, whilst at the same time nine years have passed since the opening, without creating an adequate staff to enable it to be worked as an efficient hospital possessing the best of medical and surgical treatment for acute cases, is a matter into which, perhaps, it would not be wise, even if it were just, for us to inquire definitely.

The whole place, excellent as it is in many ways, does not meet the urgent need there is in Leicester for extra hospital accommodation of the best type for the care and treatment of hundreds of the humbler citizens.

The two chairmen whom we met informed us that it had been decided to appoint a medical superintendent, who would be the responsible head of the institution, with full authority over every department and every member of the staff. This statement may come as a surprise to some of the leading men of Leicester, who were under the impression that the Guardians did not intend to pay more than £450 in salary to the medical superintendent whom they were going to advertise for, because they meant to retain £50 a year as house allowance.

We were further informed that a strong body among the Guardians hold the view that the maximum sum which any medical superintendent should receive anywhere is £500 a year. If such a feeling exists it must be amongst men who have not the knowledge or the administrative grip to justify their opinions being accepted by sober men of business as reliable or sound. It is not a matter for this or that group of Guardians, sitting round a board-room table, to lay down a fixed maximum payment for the chief official of a great Poor-Law infirmary such as North Evington was intended to be, and ought to become. The salaries paid to the medical superintendent of Poor-Law infirmaries must be based upon the market value which they represent, and until Leicester, and Leicester ratepayers, rouse themselves, and secure that the emoluments for the medical superintendent shall be sufficient to attract the best men in the Poor-Law service they will have to put up with the waste of their money. The present condition of the staff arrangements of this infirmary make this establishment a singular example of ineptitude in administration and an extraordinary and lamentable instance of what the best-administered town may be forced to suffer through the absence of that knowledge which ought to characterise the management of every institution.

The Leicester Board of Guardians are entrusted with the control of a magnificent group of buildings that only need the expenditure of a relatively small sum to make them into one of the most efficient and attractive hospitals in the Poor-Law service. All the difficulties, all the disagreements, all the criticism, and everything which is undesirable and regrettable in connection with the North Evington Infirmary in justice must be put down to the failure on the part of the Board of Guardians to realise the splendid opportunity they had. We have satisfied ourselves that everything which is regrettable and wrong at present can be speedily ended and removed by the appointment of a medical superintendent of ample experience and judgment, in whom should be vested the supreme control of the administration of the North Evington Infirmary.

Let us give an example of what we mean. We found in this infirmary that although it possesses an admirable isolation unit for the reception of septic cases arising in the maternity department, that unit has gradually been degraded from the purpose for which it was erected, at no doubt considerable cost, by putting there not only septic cases, but perfectly healthy maternity cases, whereby the health and possibly the lives of Leicester's mothers must be imperilled. Not only is this reprehensible and regrettable, but it is astounding in the sense that there is a large maternity ward in the main building, where healthy mothers free from disease and sepsis can be properly treated without any of the present risks and dangers to the health and lives of themselves and their offspring. We most earnestly hope that the Board of Guardians will make it their business immediately to take steps to confine the septic isolation block to septic cases, and that they will without delay rearrange the ample accommodation in the large lying-in ward and its appurtenances, so as to provide a delivery-room with a smaller ward for the mothers immediately after delivery, through the latter of which they will be passed in proper sequence to the lying-in ward. There is no blot on the Poor-Law administration of this country in our judgment more serious than that which omits to classify the maternity cases, to keep the healthy women from those suffering from venereal disease, and to secure that the innocent and respectable among the poor are freed from association with their less fortunate sisters in the days of their motherhood—an association which tends to demoralise the inmates and to add to the miseries of a residence in the maternity department of a Poor-Law establishment.

### Condition of the Wards and Offices.

We were gratified to find that the order, smartness, and hygienic efficiency of the wards, the baths, sanitary blocks, kitchens, linen, clothes, and store-cupboards and, indeed, every detail of the establishment under the charge of the nursing staff, were so excellent as to reflect the greatest credit upon the overworked nurses who, though relatively far too few in number, most conscientiously discharge their duties, despite some discouragement and an absence of further and adequate assistance such as their efficiency entitles them to have. They are evidently sadly overworked and unduly depressed and discouraged in their work as matters are at present. We believe that all managerial matters, including the control, the supply, and number of the nurses, would speedily be made adequate once the Guardians rise to the opportunity they have to introduce and maintain the highest efficiency throughout this establishment, by the wise selection and generous payment of a medical superintendent of full experience and proved ability.

### The Future Outlook.

We have reason to hope that when the time arrives in 1915 to reconsider the whole of the hospital accommodation throughout the country, every Poor-Law infirmary which has been gradually brought up to a thorough state of efficiency in regard to medical and surgical treatment, nursing, and general administration, so that it can claim to be up to the standard of a modern hospital, will earn the reward of efficiency by having the pauper element and stigma removed from it by its elevation to the position of a State hospital. Why should Leicester, which a close inspection of its other medical institutions reveals to be a town where the average intelligence and public spirit of the citizens approaches the maximum, and where the results they have obtained in connection with every other institution are so admirable and good, suffer possibly the most expensively constructed and structurally efficient and modern of its medical institutions to remain less than efficient for its purpose, as North Evington has been allowed to remain for the last nine years?

